# Challenges in administrative data linkage for research

**DOI:** 10.1177/2053951717745678

**Published:** 2017-12-05

**Authors:** Katie Harron, Chris Dibben, James Boyd, Anders Hjern, Mahmoud Azimaee, Mauricio L Barreto, Harvey Goldstein

**Affiliations:** 1 Department of Health Services Research and Policy, London School of Hygiene & Tropical Medicine, London, UK; 2 Institute of Geography and the Lived Environment, University of Edinburgh, Edinburgh, UK; 3 Centre for Population Health Research, Curtin University, Perth, Australia; 4 CHESS Karolinska Institutet, Stockholm University, Stockholm, Sweden; 5 Institute for Clinical Evaluative Sciences, Toronto, Canada; 6 Center of Data and Knowledge Integration for Health (CIDACS), Instituto Gonçalo Moniz, Fundação Oswaldo Cruz, CEP 41745-715 Salvador-Bahia, Brazil; 7 Graduate School of Education, University of Bristol, Bristol, UK, and UCL Great Ormond Street Institute of Child Health, London, UK

**Keywords:** Data linkage, record linkage, epidemiological studies, measurement error, selection bias, data accuracy, administrative data

## Abstract

Linkage of population-based administrative data is a valuable tool for combining detailed individual-level information from different sources for research. While not a substitute for classical studies based on primary data collection, analyses of linked administrative data can answer questions that require large sample sizes or detailed data on hard-to-reach populations, and generate evidence with a high level of external validity and applicability for policy making. There are unique challenges in the appropriate research use of linked administrative data, for example with respect to bias from linkage errors where records cannot be linked or are linked together incorrectly. For confidentiality and other reasons, the separation of data linkage processes and analysis of linked data is generally regarded as best practice. However, the ‘black box’ of data linkage can make it difficult for researchers to judge the reliability of the resulting linked data for their required purposes. This article aims to provide an overview of challenges in linking administrative data for research. We aim to increase understanding of the implications of (i) the data linkage environment and privacy preservation; (ii) the linkage process itself (including data preparation, and deterministic and probabilistic linkage methods) and (iii) linkage quality and potential bias in linked data. We draw on examples from a number of countries to illustrate a range of approaches for data linkage in different contexts.

## Background

Administrative data collected for financial or clinical management purposes contain rich, detailed information, and their great potential for research has been increasingly exploited over recent years. Linking together information across multiple data sources (e.g. health, social welfare, or employment) can further enhance existing data. As traditional methods for data collection (e.g. cohort studies and surveys) become more problematic due to high cost and low response rates or attrition, use of linked individual-level data has become an attractive alternative (Jutte et al., 2011; Pearson, 2015).

The strengths of linked administrative data are well-characterised, particularly for research requiring large sample sizes, detailed data on hard-to-reach populations, or little loss to follow-up, and for generating evidence with a high level of external validity and applicability for policy making (Holman et al., 2008). However, the limitations of administrative data are also well understood, particularly those relating to data quality and missing data (Hashimoto et al., 2014; van Walraven and Austin, 2012). For example, missing data can occur in the traditional sense, i.e. where recording is incomplete, but can also occur if a person fails to interact with a service (e.g. a school based exam or a hospital clinic) and is therefore not captured in the administrative data (Weitoft et al., 1999). Data linkage adds a further dimension: missing or inaccurate data can also be introduced if the individual’s school or hospital record could not be accurately linked due to insufficient identifying information.

Linkage of administrative data to support population-based analyses also poses a unique set of methodological challenges related to the use of personal identifiers. In many jurisdictions, the separation of linkage and analysis processes is considered as best practice for confidentiality, meaning that those conducting the linkage (often a ‘trusted third party’) only have access to a set of identifiers, whilst those analysing the linked data only have access to de-identified attribute data (Kelman et al., 2002). Although this strategy limits the risk of disclosing sensitive information about individuals, the separation of functions means that important aspects of the linkage process, which impact on the reliability of the resulting linked dataset, can be obscured from those analysing and interpreting the linked data.

This aim of this article is to improve understanding of approaches to administrative data linkage, and to provide an overview of important considerations for linking administrative data for research. We begin by considering the data linkage environment and the implications of safeguarding administrative personal data. We then provide an overview of the linkage process, including data preparation and linkage methods, and discuss how these processes may affect the linked dataset. Finally, we consider linkage quality and evaluation, and the implications of potential bias due to errors occurring during the linkage process. We draw on examples from a number of countries to illustrate approaches for data linkage in different contexts.

## The data linkage environment

A number of models for data linkage studies exist across different jurisdictions, with differing degrees of separation between linkage and analysis processes. The strictest of models involves identifiable data accessed only by a trusted third party (who conduct the linkage), whilst the research group only access de-identified attribute data required for analysis. For example, the Data Linkage Branch in Western Australia and the Centre for Health Record Linkage (CHeReL) in New South Wales receive identifiable data and use these to create anonymous ‘linkage keys’. These linkage keys are passed to researchers, who can then merge together the corresponding attribute data (e.g. clinical or service records) required for their analysis (without ever seeing any identifiers). This linkage model creates enduring links that are stored in perpetuity within the system, meaning that records do not need to be repeatedly matched for different studies (Dibben et al., 2015). Similarly, the SAIL Databank in Wales does not hold identifiers, but retains an Anonymous Linking Field (ALF), which is unique for each person and used to link multiple datasets for research (Jones et al., 2014). The Centre for Data Linkage (CDL) in Australia uses a similar model, except that identifiable data are received and linked on a project by project basis (Boyd et al., 2012).

Linkage models can vary within countries. Whilst the Manitoba Centre for Health Policy in Canada follows the same model as Australia, a different model operates at Institute for Clinical Evaluative Sciences (ICES) in Ontario. ICES is legally allowed to receive fully identifiable data in order to perform linkage, assess data quality and provide coded data to research staff within the organisation. They operate a hierarchical access policy, which means that only a specified number of people have the highest level of access to all data elements, and most researchers can only access de-identified, coded data relevant to their study. A modification to this system is the ‘split-file’ approach, used in Population Data BC, where identifiers are stripped from attribute data as soon as they are received, and stored separately, only being accessed by a certain number of programmers (who do not access the attribute data) (Dibben et al., 2015).

Full separation of identifiers and attribute data has been argued to reduce the risk of re-identification, and is a valuable tool in reassuring data providers about the security of sharing their data. However, allowing linkage and analysis to take place together provides opportunities for both in-depth evaluation of linkage quality, and methodological advances in linkage techniques (Aldridge et al., 2015; Harron et al., 2013). For example, this approach can allow alternative linkage variables that are not considered as typical personal identifiers (such as dates or diagnoses) to be incorporated into linkage algorithms or validation procedures (Hagger-Johnson et al., 2014; Harron et al., 2016).

### Accessing linked data for research

Effective data linkage environments protect confidentiality whilst facilitating research use of personal data (defined in the UK as data relating to living individuals who can be identified from those data, or from data or other information in the possession of the data controller) (Information Commissioner's Office, 2016). Producing completely anonymous datasets (where it is not possible to identify any individual) would be protective of confidentiality (Information Commissioner’s Office, 2012; Ohm, 2010). However, it is increasingly clear that full anonymization of individual-level data is virtually impossible whilst retaining sufficient granularity for research (de Montjoye et al., 2015). Instead, confidentially is preserved through a combination of (i) comprehensive data access approvals, (ii) requirements on the researcher, including training and sanctions and (iii) physical or virtual settings that restrict the possibility of re-identification of individuals or inadvertent or deliberate misuse of data. Key points of the three components of the data linkage environment are summarised in Box 1.

**Box 1.** Considerations for safe data linkage environments.

Context	Key points
Data access approvals	• Comprehensive approvals processes typically check that:
	^ There is a legal basis for data access
	^ There are appropriate security arrangements
	^ Data are used only for a specified purpose, are kept only for a specified length of time, and are not further disclosed
	^ The requesting institution has appropriate credentials
	^ The ethics of the proposed study have been properly scrutinised
Researcher requirements	• Researchers have a responsibility, often laid out in terms of use, to use data for bona fide purposes only
	• Researchers should receive regular training in information governance
	• Legal sanctions are in place where data are used inappropriately or without due care
Physical or virtual setting	• Secure physical, or virtual, locations established for the processing and linkage of personal or potentially identifiable data, characterised by:
	^ Strict access arrangements
	^ Secure data transfer processes
	^ Restricted network and/or internet access
	• Tight disclosure control procedures
	^ For example, aggregate data only, suppression of small cell sizes (e.g. < 5), k-anonymity
	• Help protect against outsider attacks or coercion
	• Provide tangible reassurance on data security to the public

Firstly, access to linked administrative data is usually overseen by an approvals panel, who consider a number of details about the data requested and the credentials of the requesting institution and researcher(s), including appropriate security measures and governance training. Approval panels are concerned with confirming the legal basis for disclosing data, and often take into account whether the proposed research is in the public benefit, and whether this benefit is outweighed by any potential risks of using the data (Dibben et al., 2015). In the UK, approvals ensure that data use meets the principles of Data Protection Act 1998, i.e. fair and lawful processing of data (Information Commissioner's Office, 2016). In Brazil, while there is legal support for the use of administrative data for research, clear evidence of advantages of data linkage for health policy is required for ethics committee approval (Farinelli et al., 2015). In Australia, record linkage stakeholders are regulated by legislation and contractual obligations relating to privacy and confidentiality.

Applications may need to be made to a number of panels, who consider different and overlapping aspects of a study. For example in the UK, a research study proposing linkage of identifiable data without consent would require applications to the data provider (e.g. the Office of National Statistics for death registration data), the trusted third party (e.g. NHS Digital, who perform linkage with hospital records), a local or national research ethics committee, and the Confidentiality Advisory Group (an independent body providing advice on applications to use confidential patient data without consent). In Australia, the federated government system means that various datasets are gathered at different tiers of administration, with different jurisdictions being responsible for different data collections. Complete population coverage can only be achieved through linkage between jurisdictions. However, there are significant differences in the access and approvals processes between each jurisdiction, often requiring researchers to obtain approval from combinations of data custodians, data linkage units and ethics committees across each legal jurisdiction.

The second component of the safe environment is the researcher. Researchers are expected to undergo information governance training before accessing data. Once data access is approved, researchers are typically required to abide by a license or contract, setting out the ways in which data may be processed. Any breaches of these contracts are subject to strict sanctions, often at the institutional level, which provide a deterrent to intentional or negligent behaviour. For example in the UK, monetary penalty notices of up to £500,000 can be levied for any data breaches. Penalties are publically available online, with implications for the reputation of both the associated researcher and their institution.

The final component of the data linkage environment is the physical or virtual setting within which data processing takes place. A safe setting (or safe haven) is a secure location where data are stored or accessed via a secure network link, which is subject to strict access arrangements. An important aspect of the safe setting is how outputs are checked (a process known as statistical disclosure control). In the context of linked administrative data, statistical disclosure control attempts to limit the risk of identification (i.e. finding out the identity of someone in a dataset) and attribution (i.e. associating information held in a record with a particular individual). A detailed description of statistical disclosure control mechanisms can be found elsewhere (Longhurst et al., 2011). A simple example of this process used in many countries is where outputs are checked to ensure that no small cell sizes (e.g. <5) are released outside of the safe setting. More sophisticated approaches include k-anonymity, which ensures that any individual cannot be distinguished from k-1 other individuals (Information Commissioner’s Office, 2012).

### Implications for research

Some argue that such extensive governance requirements can be a barrier to research, and that the harms from not using administrative data are greater than the risks (Jones et al., 2017). Firstly, data access applications often require a substantial investment of researcher time, and approvals are subject to long-delays that are difficult to align with project schedules and funding timelines (Dattani et al., 2013). Where application processes are not streamlined, the need to obtain approval from a number of different bodies can result in the same information being reviewed by different panels, each with different remits and perspectives. Secondly, physical safe settings are not typically optimal for research, as they may require travel and be restricted to set hours, and analyses often need to be repeatedly refined and reworked. Virtual safe settings are more flexible as they allow secure, remote access to data, but may be restricted by strict disclosure control procedures that, whilst appropriate for some analyses, would not be sufficient for others that require fine-grained individual-level data (e.g. time-to-event models) (Information Commissioner’s Office, 2012).

In some countries, organisations that help researchers navigate the complex requirements and facilitate access to linked administrative data for research have been established. For example, the Administrative Data Research Network (ADRN) was established as a UK-wide partnership between universities, government departments, national statistics authorities, and funders and researchers. The ADRN includes an approvals panel (who examine each research proposal), an accredited researcher training programme (the Secure Users of Research data Environment (SURE) training), and a safe setting within which researchers can access de-identified administrative data (with statistical disclosure procedures applied to any data taken outside the safe setting).

## The linkage process

### Data preparation

As many administrative datasets contain inconsistent, inaccurate or incomplete data that vary in structure, format and content, data pre-processing is a time-consuming but vital aspect of linkage (Playford et al., 2016). For example in Brazil, name is one of the main variables available for linkage of administrative data (along with sex, date of birth and municipality). Although name can be a highly discriminative variable, the number of different ways it can be structured in Brazilian datasets can be problematic: a woman with five names might have them all recorded in one dataset, but only her first and last name in another dataset. The level of data cleaning performed therefore requires careful thought, as there is a need to retain the discriminative power of individual identifiers whilst standardising variables across datasets. Heavy data cleaning can reduce the variability between identifiers and reduce the ability to distinguish one record from another (Randall et al., 2013).

Many string comparators and phonetic coding systems have been developed in order to overcome differences in the way names are recorded (Newcombe et al., 1989a, 1989b). Soundex codes, which reduce strings to four characters, are one of the most commonly used phonetic algorithms for indexing names in the English language, although other codes exist for different languages (Mortimer and Salathiel, 1995; Russell, 1918, 1922; Zahoranský and Polášek, 2010). A number of string comparators also exist, which provide a similarity score for two strings, typically based on the number of character changes needed to make the two identical (e.g. the Jaro-Winkler comparator) (Grannis et al., 2004; Winkler, 1995).

### Blocking

As the size of available administrative datasets increases, an important consideration is how to reduce the number of comparisons made between records. The analysis of large unlinked datasets can require specialist software and high performance computing, and linkage compounds the capacity issue: if every record in one dataset is compared with every record in another dataset, the total number of pairwise comparisons is the product of file sizes. Pairwise comparisons quickly become unmanageable in administrative datasets like the 100 million cohort in Brazil, which comprises detailed socio-economic data on over half of the population (114 million people at baseline) and continues to expand as new individuals are added to the register each year (Rasella et al., 2013). The problem is exaggerated further with linkage of multiple data sources.

Therefore, blocking strategies are often used, which restrict comparison pairs to those likely to match. Blocking strategies determine which records are (and are not) considered as matches, which potentially affects the overall accuracy of the linkage process. For example, blocking on a particular geographical region or location would only consider pairs of records as potential matches if they agreed on that location; any errors in this variable would prevent records from linking. Therefore, careful consideration should be given to deciding on blocking strategies, by assessing quality and completeness of each candidate blocking variable.

### Linkage methods

In some countries, a unique personal number is required for access to services and can be readily used to obtain information about individuals. For example national legislations in the Scandinavian countries have created a single unique personal identity number for each resident used in all administrative contexts; health care, judiciary, tax, military and educational systems. Such identifiers practically make it possible to link data from many different administrative sources with marginal error (Ludvigsson et al., 2009). Linkage with these unique personal identifiers is so accurate that data can be pooled from different countries to create very large study populations, thereby enabling longitudinal studies of rare medical conditions with a cohort approach (Olsen et al., 2010).

However in many other countries, unique identifiers for linkage across sources are not available, because unique identifiers that do exist are domain specific and have been created by administrative organisations for their own purposes, and may operate at different levels of jurisdiction (Ludvigsson et al., 2009; United National Economic Commission for Europe, 2007). For example in the UK, the National Insurance number is used by Her Majesty’s Revenue & Customs and the Department of Work and Pensions for employment and taxation data; the National Health Service (NHS) number is used for health services in England, and the Community Health Index (CHI) number is the primary health identifier in Scotland; none of these are reliably used in education data. In Ontario and Manitoba, encoded provincial health card numbers that are used to link health data are not used in other government departments such as social care, education or immigration (Chiu et al., 2016). Similarly in Brazil and Australia, different numbers are used for different administrative purposes (e.g. identification, employment, taxation, social protection, and health).

In the absence of a unique identifier, linkage needs to balance the risk of missed-matches (failing to link records belonging to the same individual) with false-matches (erroneously linking records belonging to different individuals) (Table 1). There are two broad approaches to data linkage: deterministic and probabilistic. Both methods rely on finding agreement on a set of common identifiers such as name, date / place of birth, and address. In practice, linkage projects often use a combination of deterministic and probabilistic methods, with algorithms developed in an iterative process of trial and error, involving manual review and estimation of linkage error rates (Roos et al., 1986).

**Table 1.** Linkage error.

	Match status	
	Match (pair from same individual)	Non-match (pair from different individuals)
Link status		
Link	Identified match	False-match
Non-link	Missed-match	Identified non-match

#### Deterministic linkage

Deterministic linkage uses a set of pre-determined rules to classify records as belonging to the same or different individuals. For example in the UK, hospital admission records for the same individual over time are linked together using a three-step deterministic algorithm based on combinations of NHS number, date of birth, sex and postcode (Hagger-Johnson et al., 2015). Deterministic methods are typically prone to missed-matches, as any recording errors or missing values can prevent a set of identifiers from agreeing. Conversely, false-match rates are typically low, as records belonging to different individuals are unlikely to agree on a complete set of identifiers by chance (Grannis et al., 2002).

#### Probabilistic linkage

Probabilistic methods are arguably more suited to linkage of error-prone administrative data, which can also be subject to changes over time (e.g. addresses) (Fellegi and Sunter, 1969; Newcombe et al., 1959; Sayers et al., 2015). In probabilistic linkage, a match weight is assigned to each pair of records, with higher weights indicating a greater likelihood that the pair is a true match. Where identifiers agree, a positive contribution is made to the match weight; disagreement contributes a penalty to the weight. In the simplest case, each identifier contributes separately to the match weight, taking into account the discriminative value of each identifier, so that, for example, agreement on date of birth would contribute more evidence of a match than agreement on sex.

In the standard Fellegi–Sunter probabilistic procedure, match weights are derived from two conditional probabilities: the m-probability (the probability that an identifier agrees given records belong to the same individual) and the u-probability (the probability that an identifier agrees given records belong to different individuals). The u-probability represents the frequency of values for each identifier, i.e. the probability of chance agreement on sex is ½; the probability of chance agreement on month of birth is 1/12, and so on. M-probabilities represent the error rate in a particular identifier. For example, if sex were miscoded in 5% of record pairs, the m-probability would be 0.95. These probabilities are typically estimated via a statistical model, and the overall match weight is calculated as a function of these probabilities, usually the ratio log_2_(*m*/*u*) for each identifier, summed across all identifiers (Brown et al., 2017). Adaptations to standard match weight calculation include frequency-specific match weights, which assign greater weights to less common identifier values and thus provide greater discrimination between matches and non-matches (Zhu et al., 2009).

Record pairs are classified as links or non-links depending on whether the corresponding match weight reaches a cut-off threshold. Often, two thresholds are chosen: pairs with weights above the upper threshold are classified as links; pairs with weights below the lower threshold are classified as non-links; those in the middle are inspected further (e.g. through manual review). Choice of threshold values is important, as adjusting the thresholds changes the balance between the number of false-matches and missed-matches (Krewski et al., 2005). However, choosing optimal thresholds is not straightforward, and is often a subjective process based on manual review of record pairs, guided by plotting the distribution of match weights (Blakely and Salmond, 2002; Dusetzina et al., 2014). If manual review is not feasible, e.g. due to a lack of resources or too large a dataset, a single optimal threshold may be chosen by calculating quality measures at a number of different threshold values and comparing these to levels of acceptable error for a particular study (Christen and Goiser, 2007).

Many linkage systems often use a combination of deterministic and probabilistic approaches. Deterministic methods are computationally inexpensive relative to probabilistic methods and are easier to implement, but may not achieve sufficient linkage quality.

#### Privacy preserving linkage

There are some situations in which identifiers cannot be released for linkage. For example, the Office for National Statistics Beyond 2011 programme involved linkage of information from different government departments on all individuals in England and Wales to support the UK Census, and a decision was made to handle only non-identifiable data to maintain a high level of data security. The programme therefore explored privacy preserving record linkage (known as PPRL) for linking encrypted identifiers (Abbott et al., 2015). Encryption transforms identifiers (such as date of birth or name) into hashed values in order to avoid re-identification of individuals. The challenge in the adoption of privacy preserving methods is achieving high levels of privacy protection without negatively impacting on performance and linkage quality (accuracy of results). One of the limitations of encrypted identifiers is that, by design, similar identifiers look very different once encryption has taken place. For example, a hash function may transform the name “John” to the string ‘8C17A3BB4CAF719D165097900B390161’ and the name ‘Jon’ to ‘861A421C1A05E0E8FA24A1534159691F’. Only one character differs in the original identifiers, yet the hashed values are completely different. This complicates the process of assessing the *similarity* between identifiers on different records.

Such problems can be overcome through the use of ‘match-keys’, which take elements from each identifier (e.g. first letter of first name, first letter of second name, day of birth and postcode prefix), or Bloom filters, which decompose a string into bigrams (2-character strings) and map these bigrams to a specific position in a binary array. Bloom filters are more complex data structures than standard hashing functions, and although they preserve anonymity, can be compared using a similarity index such as the Dice coefficient (Schnell et al., 2009). In Brazil, software using encrypted data via Bloom filters has been developed to link the 100 million cohort (using Spark) (Pita et al., 2015). Australia have also progressed probabilistic linkage using Bloom Filters to supplement existing linkage systems. A recent project successfully verified PPRL using Bloom filters in terms of privacy, scalability, error tolerance and security, using real-world data from New South Wales and Western Australia.

#### Alternative linkage methods

Although traditional methods rely on personal identifiers for linkage, other auxiliary variables can provide further evidence about linkage probabilities. For example, a measure of height in one file and a measure of weight in the other could potentially provide information about the likelihood of a true match. Indirect identifiers, such as clinical information, have also been successfully incorporated within linkage algorithms and have the potential to reduce disclosiveness within linkage (Harron et al., 2016; Setoguchi et al., 2014). One approach that utilises such auxiliary variables is ‘prior informed imputation’, which treats linkage as a missing data problem, incorporating information on these variables at the stage of model fitting and performing an imputation procedure using the probabilistic weights assigned to ‘candidate’ records as Bayesian priors (Goldstein et al., 2012). The advantage of this method is that it exploits relationships between identifiers and non-identifying variables (Goldstein and Harron, 2015; Harron et al., 2014). A number of other Bayesian models for linkage have been explored, but are not yet widely used due to a number of required assumptions about distribution of errors, file structure and model specification (Tancredi and Liseo, 2011).

### Implications for research

The nature of the data to be linked will determine whether a large-scale linkage system is established (requiring a dedicated IT infrastructure and support), where linked datasets are produced in a ‘one size fits all’ manner, or whether ad-hoc linkage can be achieved, tailored to a specific research question (Jones et al., 2014). In each of these scenarios, choices will need to be made regarding data cleaning procedures, strategies for blocking, and linkage methods. Ideally, these choices are based on contextual knowledge about the quality and quantity of identifiers in the underlying datasets, which may come from both the data provider and the researcher. However, choices may also be restricted by the availability of identifiers, e.g. if governance requires that only encrypted identifiers can be released. PPRL remains a contentious issue, as any errors in original identifiers are embedded within encrypted identifiers, meaning that this approach is less flexible and more difficult to evaluate than linkage using unencrypted identifiers. Achieving a balance between data protection and accuracy and usability of the resulting linked dataset is an ongoing area of research (Vatsalan et al., 2013).

Sharing of information about each step of the linkage process between data providers, linkers and analysts, can help improve transparency and increase understanding of the reliability of the linked data (Gilbert et al., 2017). For example, Statistics Canada have published a Record Linkage Project Process Model, which describes common practices for linkage within the Agency (Sanmartin et al., 2017). ICES in Ontario produce a ‘Linkability Report’ for each of their data holdings, which provides the number and percentage of linked records (by type: deterministic or probabilistic) and unlinked records by year.

## Evaluating linkage quality

### Linkage error

Linkage error arises when pairs of records are incorrectly classified (Table 1). False-matches occur when records from different individuals link erroneously. Missed-matches, where records from the same individual fail to link, occur in data where identifiers are prone to misreporting (e.g. typographical errors), changes over time (e.g. married women’s surnames; addresses) or missing values. Linkage errors in administrative data are inevitable due to the imperfect and transient nature of identifiers, and even small amounts of linkage error can result in substantially biased results (Neter et al., 1965).

Missed-matches can result in a loss of generalisability, or selection bias, if particular subgroups of records are more or less likely to link (non-random or differential linkage error) (Bohensky et al., 2010; Ford et al., 2006; Lariscy, 2011). Depending on the data source, studies have found that data quality varies according to a number of characteristics including age, sex, ethnicity and health status (Bohensky, 2015). This can lead to, for example, lower match rates in more vulnerable or deprived populations.

False-matches are a further challenge. When records from two different individuals are linked together, associations between variables can be diluted or spurious associations created. When a record is linked but no link should have been made (e.g. linking a survivor to a mortality record), this can have implications for prevalence estimates (such as overestimating a rate). If false matches depend on individual characteristics (e.g. sex, because of maiden/married name inconsistencies) this may lead to biased estimates of association, e.g. if sex is related to both the exposure and outcome of interest.

### Measuring linkage error

Many linkage studies report the proportion of records that were linked, i.e. the match rate. Other frequently reported measures of linkage quality include sensitivity and specificity, and positive and negative predictive values, which are directly related to rates of false- and missed-matches (Christen and Goiser, 2005; Ferrante and Boyd, 2012). However, these measures in themselves do not provide information on how results of analyses might be affected in terms of bias, and are not always relevant. For example, match rate is only helpful if you know how many records from a particular dataset should be linked.

Box 2 summarises several methods for evaluating linkage quality, including comparisons with gold-standard data, post-linkage validation, comparisons of linked and unlinked data, and sensitivity analyses (Harron et al., 2017).

**Box 2.** Evaluating linkage quality.

Approach	Key points
‘Gold standard’ or reference data	• Data where the true match status is known, used to test linkage algorithms and estimate rates of linkage error.
	• Typically based on a subsample of records that have been manually reviewed, an additional data source with complete identifiers, a representative synthetic dataset, or external reference rates for the population of interest
	^ For example, comparison of mortality rates based on linkage of death registrations versus national figures (Schmidlin et al., 2013) or comparison of infection rates within a subset of validated data (Harron et al., 2013, Paixao et al., in press).
Post-linkage data validation	• Used to estimate minimum false-match rates by identifying implausible scenarios within the data.
	^ For example, linkage of a hospital admission record following a known date of death could indicate a false-match; as could linkage of multiple death records to a single census record (Blakely and Salmond, 2002; Hagger-Johnson et al., 2014).
Sensitivity analyses	• Used to assess the extent to which results vary according to different linkage criteria.
	• Could involve changing the linkage algorithm or changing the threshold within probabilistic linkage, and re-running analyses to evaluate any impact on results (Lariscy, 2011).
	^ For example, comparing results over a range of match weights could help identify the direction of the effect of linkage errors on outcomes of interest (Moore et al., 2014).
Comparing characteristics of linked and unlinked data	• Used to identify any differences in linkage rates for different subgroups of individuals.
	^ For example, comparing rates of preterm birth in linked and unlinked maternity records (Ford et al., 2006; Harron et al., 2016).
	• Where not all records are expected to match, distributions of variables in the linked data can be compared to external sources (e.g. age and/or ethnic group distributions from national census data) to explore any evidence of selection bias (Harron et al., 2016).

### Implications for research

Linkage error can threaten the reliability of results based on analyses of linked administrative data. However, effective communication between data providers, linkers and analysts allows sharing of information that enables the quality of linkage to be evaluated (Gilbert et al., 2017). Analysis strategies can then be based on an understanding of the data linkage processes, the context of the data itself and the research question to be addressed.

The type of evaluation conducted will depend on the context of the data environment (Harron et al., 2017). For example, evaluation of linkage using a gold-standard dataset is usually performed by those conducting the linkage, since researchers themselves rarely have access to identifiable data. In contrast, post-linkage validation, sensitivity analyses and comparison of characteristics of linked and unlinked records can be performed by the researcher, given the data linker provides certain non-sensitive information about the linkage process. Information that should be passed on to researchers to facilitate these evaluations include meta-data on the quality of each link (such as the decision-rule or match weight), and record-level or aggregate characteristics of unlinked records (to identify potential sources of bias).

For example, the body performing most linkage of hospital records for the NHS in England (NHS Digital) provide data users with a match rank for each linked record that indicates which identifiers were used for a particular match. The Institute of Clinical Evaluative Sciences in Ontario provide researchers with a linkage report, which summarises the linkage strategy and outcomes for each linkage step; linkage weights can be added to each record in the linked data. This information is helpful for researchers to understand exactly how decisions about each record were made, to evaluate the quality of each link, and to take into account potential biases.

Study designs should be informed by information on the quality of linkage, and can be optimised to account for potential bias due to linkage error, or uncertainty in linkage between data sources. For example, consider an ‘informative’ linkage, aiming to ascertain case-status by linking to a registry dataset (such as death notifications or infection surveillance). In this scenario, linkages with high positive predictive value lend themselves to case–control study designs, which require certainty that linked records really do represent true cases, but do not necessarily require all possible matches to be identified (Paixao et al., in press). This strategy requires discussion between researchers and data linkers, so that criteria for defining records as certain links and certain non-links can be agreed upon. On the other hand, linkages with high levels of linkage are more relevant to cohort study designs that prioritise high sensitivity to provide reliable prevalence estimates. In the latter case, analyses can also incorporate inverse-probability weights (e.g. from survey methodology), to provide values for records that could not be accurately linked. Further methods such as prior informed imputation or multiple imputation can also be used where there is uncertainty about the correct link, provided certain non-sensitive characteristics that predict linkages are shared with researchers (Harron et al., 2014).

## Remaining challenges and future directions

While many of the technical challenges of safe data linkage environments have been overcome, there are situations where significant legal and administrative challenges remain (Harron et al., 2015). These, in turn, impact on data availability and accessibility for research and policy development. Although some jurisdictions adopt approaches for timely and cost-effective access to linked data (e.g. those in Ontario, Wales and Australia where linkage keys can be held in perpetuity), others are restricted by the ‘link and destroy’ model, where linked data cannot be reused. A lack of streamlined approval processes also contributes to inefficient processes for data access.

There are a number of areas of ongoing research in facilitating access to data once it has been linked. For example, data perturbation adds noise to data so that the risk of re-identification is reduced to within specified limits, i.e. by fixing the probability that a record corresponds to the target individual. This technique retains the statistical properties of data for analyses and requires that analysts adjust for the added noise using a measurement error model. Alternatively, synthetic data allow researchers to test out analyses on a dataset that mimics the structure of real data but that does not correspond to real individuals. This allows researchers to explore potential modelling strategies prior to analysing the original data, thus reducing the time spent within a safe setting (Dennett et al., 2015). However, selecting appropriate models for a particular analysis relies on the correct structure being identified in the synthetic data, otherwise model estimates may be biased.

The need to balance both privacy (for the individual) and quality (for research purposes) of linked data is a priority for research in data linkage methods. The dynamic, error-prone and incomplete nature of administrative data makes a certain level of linkage error inevitable, and this is compounded when data are required to be anonymised before linkage. Developing methods to adjust for biases arising for linkage error is therefore vital for producing robust evidence to inform policy.

Bridging the gap between linkage and analysis is a major challenge for progress in the area of linkage quality. Researchers often struggle to obtain the information they need to evaluate linkage and to developing methods to account for any potential bias due to linkage error (Jorm, 2015). Recently published guidelines on the information that should be shared between data linkers and researchers are an important step towards increasing the reliability of research using linked administrative data (Gilbert et al., 2017). Sharing of this information can support transparent reporting of studies using linked administrative data (Benchimol et al., 2015). As methodologies continue to evolve to address issues of data security and quality, there is an ongoing need to evaluate the most effective ways of sharing this information.
